# Relationship between dynamic changes of peri-procedure anxiety and short-term prognosis in patients undergoing elective percutaneous coronary intervention for coronary heart disease: A single-center, prospective study

**DOI:** 10.1371/journal.pone.0266006

**Published:** 2022-04-01

**Authors:** Yao-yao Hu, Ya-jing Cai, Xin Jiang, Fang-ying Mao, Jing Zhang, Lin Liu, Qing Wu, Xiao-hua Wang

**Affiliations:** 1 Department of Cardiology, The First Affiliated Hospital of Soochow University, Suzhou, Jiangsu Province, P.R, China; 2 Department of Cardiology, Changshu NO.1 People’s Hospital, Suzhou, Jiangsu Province, P.R, China; 3 Nursing Department, The Affiliated Wuxi People’s Hospital of Nanjing Medical University, Wuxi, Jiangsu Province, P.R, China; 4 School of Nursing, Soochow University, Suzhou, Jiangsu Province, P.R, China; Baylor Scott and White, Texas A&M College of Medicine, UNITED STATES

## Abstract

**Background:**

Patients with coronary heart disease (CHD) often experience anxiety, but the current studies on anxiety mostly focused on a certain point in time. Therefore, this study aimed to investigate the dynamic changes of peri-procedure anxiety, status of post-procedure quality of life, and cardiovascular readmission rates in patients with CHD who undergoing elective percutaneous coronary intervention (PCI), and to analyze the influence of peri-procedure anxiety on quality of life and readmission rate after PCI.

**Methods:**

This prospective study was conducted at Changshu NO.1 People’s Hospital. A total of 220 patients with CHD undergoing elective PCI were selected as study subjects. The general information, clinical data, anxiety, quality of life and readmission of patients were collected. Multivariate linear regression was used to examine the effect of peri-procedure anxiety on quality of life, and multivariate logistic regression was used to analyze the influence of peri-procedure anxiety on readmission rate.

**Results:**

This study showed the anxiety scores at hospitalization appointment(T1), 3 days before procedure(T2), 1 day before procedure(T3), 1 day after procedure(T4) were 57(55,61),64(61,68),54(51.58), and 54(50,60), respectively. And, at 3 months and 6 months after PCI, the scores of Seattle Angina Questionnaire (SAQ) were 346.61(319.06,366.52) and 353.34(334.18,372.84) respectively. During 6 months follow-up, 54 cases were readmitted, with a readmission rate of 25.5%. Statistical analysis showed that T1 with anxiety (*P* = 0.002) and T2 with anxiety (*P* = 0.024) were independent risk factors for treatment satisfaction at 3 months after PCI. Anxiety in T4 (*P* = 0.005) was an independent risk factor on the angina frequency at 6 months after PCI. T2 with anxiety (B = 1.445, *P* = 0.010, 95%CI:1.409–12.773) and T4 without anxiety (B = -1.587, *P* = 0.042, 95%CI:-0.044–0.941) were risk factors affecting readmission for cardiovascular reasons within 6 months.

**Conclusion:**

Patient anxiety at T1 and T2 affects the treatment satisfaction dimension of the SAQ at 3 months after PCI, and anxiety at T4 affects the angina frequency dimension of the SAQ at 6 months after PCI. Anxiety at T2 and no anxiety at T4 increase short-term readmission rates. In the future, interventions should be strengthened at various time points in the peri-procedure period to improve post-procedure rehabilitation effect.

## Introduction

Coronary heart disease (CHD) is a kind of heart disease caused by coronary artery atherosclerosis, which causes stenosis or obstruction of the vascular lumen, resulting in myocardial ischemia, hypoxia or necrosis [1]. In recent years, cardiovascular disease (CVD) has been become the most important cause of death in the world [2, 3]. At present, percutaneous coronary intervention (PCI) has become one of the most important means of coronary heart disease treatment due to its advantages of high safety, minimal injury, less pain and shorter hospital stay. PCI can be classified as emergency PCI or elective PCI according to the timing of procedure [4]. Elective PCI refers to the treatment strategy that patients have no indication of emergency PCI and doctors rationally arrange PCI based on the characteristics of their conditions, it is suitable for patients with stable angina and low-risk non-ST-segment elevation acute coronary syndrome [5]. According to the statistics of China cardiovascular disease quality control center in 2019, the number of PCI cases in China has increased from 500000 in 2014 to 915000 in 2018, ranking first in the world.

Although PCI significantly reduced the symptoms of CHD, reduced mortality, and improved the quality of life [6], some patients still had the risk of major adverse cardiovascular events (MACE) and readmission after procedure. In one study, the readmission and mortality rates in elderly patients with stable angina were 0.48% and 0.02%, respectively, 2 days after elective PCI, and 9.6% and 0.22%, respectively, within 30 days after elective PCI [7]. However, the prognosis of patients with CHD is related to many factors, and negative emotion is one of the influencing factors. Procedure is a very threatening stressor, which often leads to a strong psychological stress response before procedure, the most typical of which is anxiety [8], which can be as high as 34.7% [9]. Anxiety can lead to the increase of sympathetic nerve excitability, catecholamine level and the release of procoagulant substances in large quantities, which can accelerate the heart rate and induce or add cardiovascular events such as angina and myocardial infarction [10, 11].

A survey showed that 37.6% of 250 patients with CHD underwent PCI had anxiety [12]. Another survey of anxiety in 203 patients with coronary heart disease who underwent PCI found that the incidence of anxiety before PCI was 22.2%, and the incidence of anxiety and depression was 16.7%, while the incidence of anxiety one week after PCI was 9.9%, and the incidence of anxiety and depression was 14.8% [13]. It can be seen that anxiety occurs in different time periods before and after PCI. Anxiety and CHD are causes and effects on each other, patients with CHD are prone to anxiety, and anxiety seriously affects the prognosis of patients with CHD [14, 15]. Therefore, it is very important to identify and intervene patients with anxiety.

At present, studies on the anxiety of patients with CHD mostly focus on a certain time point before or after procedure, the period from appointment to procedure is an important part of the peri-procedure period for patients with CHD, many patients develop psychological problems during this period. What level does their anxiety reach and if it affects the prognosis? It has not been reported at home and abroad. And there is a growing interest in how to alleviate the anxiety associated with procedure and improve the patients’ psychological coping skills. So, the aims of this study were as follows: (1) to investigate the dynamic changes of peri-procedure anxiety in patients with CHD underwent elective PCI; (2) to analyze the influence of peri-procedure anxiety on post-procedure quality of life and readmission rate for cardiovascular reasons.

## Materials and methods

### Study design

**Fig 1:**
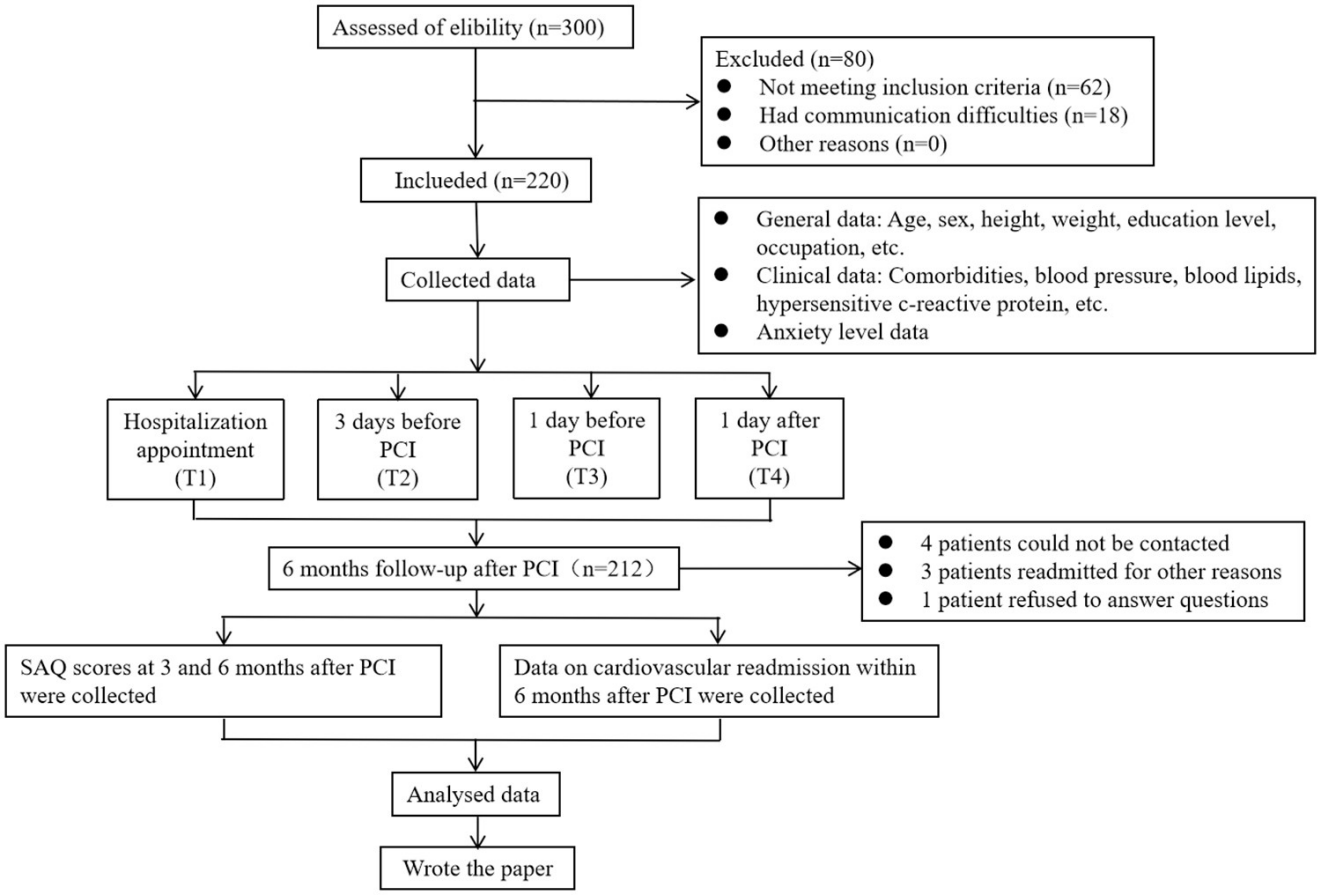
Flow chart of recruitment and follow-up for the entire study. PCI = percutaneous coronary intervention; SAQ = Seattle Angina Questionnaire.

This prospective single-center study was conducted at Changshu NO.1 People’s Hospital from December 2019 to December 2020. Changshu NO.1 People’s Hospital is a tertiary hospital, and the Department of Cardiology is a key clinical specialty in Changshu, with 60 open beds. In this study, we mainly collected the anxiety data of the patients during the peri-procedure period, and followed up the patients at 3 and 6 months after PCI to collect the quality of life data and readmission data for cardiovascular reasons, so as to explore the influence of peri-procedure anxiety level on short-term prognosis. Written informed consent was obtained from all participants, and every participant received detailed oral or written presentations prior to signing the informed consent form. All participants were guaranteed confidentiality upon receipt of the questionnaire. This study was based on the declaration of Helsinki and was approved by the Ethical Committee of Changshu NO.1 People’s Hospital (approval number 201909). The protocol of this study has been registered in Chinese Clinical Trials Registry (registration number ChiCTR2000035801). A total of 300 study subjects were recruited, of whom 62 did not meet the inclusion criteria and 18 had communication difficulties, leaving 220 subjects who met the study criteria and agreed to participate in the study. During the 6-month follow-up, 4 cases could not be contacted, 3 were readmitted for other reasons, and 1 refused to answer questions. The final 212 subjects were included in the statistical analysis (Fig 1).

### Participants

Patients were included in our study in our study have to meet the following criteria: (1) had age 18 years old and above; (2) met the diagnostic criteria of CHD in Internal Medicine (9th Edition) [16]; (3) received elective PCI treatment for the first time; (4) agreed to participate in the study and signed informed consent; and (5) the patient had no disease that affected their survival time, such as malignant tumor, severe liver and kidney disease. Participants were excluded if they (1) had an acute infection recently; (2) had mental illness or cognitive impairment; and (3) developed other conditions during follow-up that affect survival.

According to the requirement of sample size to explore the influencing factors of relevant variables, the sample size should be at least 15–20 times the number of variables [17]. In this study, there were about 10 influencing variables, so a sample size of 150–200 cases was required. Considering a sample loss rate of 20%, it was decided to include 220 subjects in this study to improve the validity of the questionnaire measurement.

Participants were recruited at Changshu NO.1 People’s Hospital, Suzhou, China. Recruitment was done by advertising the study on the hospital website or on bulletin boards. The first subject was recruited on 30 December 2019, with the entire recruitment process lasting from December 2019 to March 2020. A total of 300 patients with CHD treated with elective PCI were identified, of which 62 did not meet the inclusion criteria and 18 had communication difficulties, thus leaving 220 patients who met the study criteria to participate in this study.

### Data collection and measurement

Data collection by centrally trained research team members (including 3 mid-level or higher nursing staff, 3 nursing graduate students and 1 doctor with deputy senior title in cardiology department). Because the average time from appointment to procedure is about 1 week in our hospital, when collecting the data of peri-procedure anxiety, we take the time of hospitalization appointment as T1, the waiting period at home (about 3 days before procedure) as T2, 1 day before procedure (when admission was completed before procedure) as T3, and 1 day after procedure as T4.

After explaining the purpose of the study to the participants, the Self-Rating Anxiety Scale (SAS) was distributed in the ward demonstration room at the time of T1 and T4. The participants or their families were instructed to fill in and take back the SAS on the spot. A general information questionnaire was distributed in the demonstration room at the time of T3, the questionnaire was completed by the participants or their families, and members of the research team used the Delano Healthcare Information Platform to search for clinical data to improve the questionnaire and guide them to fill in the SAS. At the time of T2, the research team members asked the patients about anxiety by telephone and completed the SAS. The short-term prognosis is defined as the occurrence of MACE in patients with CHD after 3 or 6 months of PCI [18], in this study, we collected the quality of life in 3 to 6 months after PCI and the readmission for cardiovascular reasons within 6 months. Readmission was defined as an unexpected and unpredictable readmission due to the same or related illness after the previous hospitalization [19, 20]. In this study, readmission referred to readmission due to cardiogenic causes such as hypertension, angina pectoris, myocardial infarction, arrhythmia and cardiac insufficiency. At 3 and 6 months after PCI, cardiologists followed up the patients by telephone to inquire about their health status, complete SAQ, and collect patient readmission data, respectively.

A self-designed questionnaire was used to collect general information on patients, including demographic information (age, gender, education, employment status, smoking history, comorbidities, etc.) and clinical information (including left ventricular ejection fraction (LVEF), hyper-sensitive c-reactive protein (hs-CRP), total cholesterol (TC), triglycerides (TG), high-density lipoprotein cholesterol (HDL-C), low-density lipoprotein cholesterol (LDL-C), etc.). Our research team generally used the Delano Healthcare Information Platform or medical records to view general information about patients.

The Self-rating Anxiety Scale (SAS) was used to measure anxiety, which was developed by Chinese professor Zung [21]. According to the results of the Chinese norm [22], the cut-off value of the SAS standard score is 50, 50–59 points is mild anxiety, 60–69 is moderate anxiety, and more than 69 is severe anxiety. The reliability coefficient and validity coefficient of the total score were 0.93 and 0.86 respectively. Cronbach’s α of this tool was 0.82 in this study.

The Seattle Angina Questionnaire (SAQ) was used to assess the specific functional status and quality of life of patients. The scale was developed by American scholar Spertus [23], with 19 items and 5 dimensions. The 5 dimensions are physical limits (PL), angina stability (AS), angina frequency (AF), treatment satisfaction (TS) and disease perception (DP). Each item is scored from 1 to 6 points, the dimension standard score is the sum of the actual scores of the dimension items, and then the actual score is converted into the standard score. The conversion formula is: standard score (%) = (actual score -lowest score of the dimension)×100/(the highest score of the dimension–the lowest score of the dimension). The higher the score, the better the patient’s quality of life and body function. The Chinese version of this scale had good reliability and validity [24], and the Cronbach’s α coefficient in this study was 0.85.

### Statistical analysis

Data were analyzed by another researcher who was blinded to the allocation using and all statistical analyses were performed in SPSS ver.23.0 (SPSS, Inc., Chicago, IL). Categorical variables were presented as frequencies and percentages. For continuous data, means and standard deviations were calculated for normally distributed variables. For data not-normally distributed median, 25^th^ and 75^th^ percentile were used. The distribution of continuous variables across subgroups of categorical variables was compared using Student’s t-test and non-parametric test was used for the skewed data between groups. The relationship between categorical variables were analysed using the Chi-square test or Fisher’s exact test. The effect of peri-procedure anxiety on quality of life was analyzed by multivariate linear regression, and the effect of peri-procedure anxiety on readmission rate was analyzed by multivariate logistic regression. For all analyses, missing data were transformed by mean imputation. Throughout the analysis, a p-value of less than 0.05 means the difference is statistically significant.

## Results

During the follow-up period, 4 cases could not be contacted, 3 cases were hospitalized for other illnesses, 1 case refused to answer questions, and eventually, the data of 212 patients were statistically analyzed.

### Sample characteristics

The average age of 212 patients was 65.42±9.96 years old, and 136 (64.2%) of them were males, and most of them were below junior high school. The characteristics of the subjects are detailed in Table 1.

**Table 1:** Sample characteristics.

Characteristic	Total(n = 212)
Age (Years)^a^	67 (59,73)
Gender^b^	
Male	136 (64.2)
Female	76 (35.8)
Level of education^b^	
Junior high school and below	147 (69.3)
Senior high school and above	65 (30.7)
Job^b^	
Yes	52 (24.5)
No	160 (75.5)
History of primary disease (Yes) ^b^	
Hypertension	103(48.6)
Diabetes	41 (19.3)
Family history of CHD	7 (3.3)
Implantable stent^b^	
Yes	112 (52.8)
No	100 (47.2)
History of smoking (Yes)^b^	103 (48.6)
Type of drugs taken (type)^b^	
≤3	121 (57.1)
4–5	85 (40.1)
≥6	6 (2.8)
BMI (kg/m^2^)^a^	23.44 (21.90,25.95)
LVEF (%)^c^	61.70±1.98
Hs-CRP (mg/L)^c^	5.28±0.45
TC (mmol/L)^c^	3.79±0.89
TG (mmol/L)^c^	1.65±0.31
HDL-C (mmol/L)^c^	1.15±0.24
LDL-C (mmol/L)^c^	1.71±0.12

^a^ The values are given as the median and 25^th^ and 75^th^ percentile.

^b^ The values are given as n (%).

^c^ The values are given as the mean and standard deviation.

BMI: body mass index, LVEF: left ventricular ejection fraction, hs-CRP: high sensitivity C-reactive protein, TC: total cholesterol, TG: triglyceride, HDL-C: high-density lipoprotein cholesterol, LDL-C: low-density lipoprotein cholesterol.

### Status of peri-procedure anxiety

The results showed that the anxiety score of T1 was 57(55,61), 140 cases were mild anxiety. The anxiety score of T2 was 64(61,68), the patients with mild, moderate and severe anxiety were 21 cases, 152 cases and 38 case respectively. The anxiety score of T3 was 54(51,58), the patients with mild, moderate and severe anxiety were 148 cases,30 cases, and 12 cases respectively. The anxiety score of T4 was 54(50,60), mild, moderate and severe anxiety were 105 cases, 39 cases and 18 cases respectively. Table 2 shows the details.

**Table 2:** Status of peri-procedure anxiety.

Point of time	Anxiety score (n = 212)^a^	Anxiety level^b^		
		Mild*	Moderate*	Severe*
T1	57(55,61)	140 (66.0)	62 (29.2)	6 (2.8)
T2	64(61,68)	21 (9.9)	152(71.7)	38 (17.9)
T3	54(51,58)	148 (69.8)	30 (14.2)	12 (5.7)
T4	54(50,60)	105 (49.5)	39 (18.4)	18 (8.5)

^a^ The values are given as the median and 25^th^ and 75^th^ percentile.

^b^ The values are given as n (%).

* A score of 50 to 59 was defined as mild anxiety, 60–69 was defined as moderate anxiety, a score above 69 was defined as severe anxiety [21, 22].

### SAQ scores at 3 months and 6 months after PCI

The results of this study showed that the SAQ scores were 153.42(129.78,180.35), 346.61(319.06,366.52) and 353.34(334.18,372.84) before, 3 and 6 months after PCI, respectively. There were statistically significant differences in scores among all dimensions (All *P*<0.001). The results are shown in Table 3.

**Table 3:** SAQ scores at before, 3 and 6 months after PCI.

Dimensions	Seattle Angina Questionnaire scores (n = 212)^a^			*χ* ^*2*^	*P* value^b^
	pre-procedure	3 months after PCI	6 months after PCI		
Physical limits	62.22(53.33,71.11)	61.11(53.33,77.78)	73.33(57.78,77.78)	38.35	<0.001**
Anginal stability	0.00(0.00,25.00)	100.00(75.00,100.00)	75.00(75.00,100.00)	311.51	<0.001**
Anginal frequency	30.00(20.00,40.00)	70.00(60.00,80.00)	70.00(60.00,80.00)	330.44	<0.001**
Treatment satisfaction	0.00(17.64,35.29)	52.94(52.94,64.71)	52.94(52.94,64.71)	306.42	<0.001**
Disease perception	33.33(16.67,50.00)	66.67(58.33,75.00)	75.00(66.67,75.00)	239.30	<0.001**
Total score	153.42(129.78,180.35)	346.61(319.06,366.52)	353.34(334.18,372.84)	316.76	<0.001**

^a^ The values are given as the median and 25^th^ and 75^th^ percentile.

^b^ Friedman test was used to compare the quality of life at pre-procedure,3 months after PCI and 6 months after PCI.

**P< 0.01.

### Status of readmission at 6 months after PCI

During the follow-up period of half a year, 54 cases were readmitted with a readmission rate of 25.5%, including 26 cases of hypertension (48.15%), 14 cases of recurrent angina pectoris (25.93%), 6 cases of cardiac insufficiency (11.11%) and 8 cases of arrhythmia (14.81%).

### Relationship between peri-procedure anxiety and post-procedure SAQ scores

Statistical analysis showed that the anxiety level at Time points T1, T2 and T3 were correlated with TS and DP dimensions of life quality 3 months after PCI, while the anxiety level at time point T4 was correlated with AF dimensions of life quality 6 months after PCI. After adjusting for other confounding factors, anxiety at T1 and T2 were the independent risk factor on the Treatment satisfaction dimensions of SAQ at 3 months after PCI (*R*^*2*^ = 0.564, *F* = 621.272), and anxiety at T4 was an independent risk factor on the Anginal frequency dimensions of SAQ at 6 months after PCI (*R*^*2*^ = 0.629, *F* = 370.106). The results are shown in Tables 4 and 5 (Only statistically significant items were listed). To understand the relationship between readmission and quality of life, we conducted statistical analysis on readmission and SAQ total score of patients 3 months and 6 months after PCI as well as between each dimension. The results showed that readmission and SAQ total score of patients 3 months and 6 months after PCI as well as between each dimension had no statistical significance. The results are shown in Table 6.

**Table 4:** Univariate analysis of influencing factors on the various dimensions of SAQ (only the meaningful parts are listed).

SAQ Dimension	Variable	3 months after PCI		6 months after PCI	
		Test statistics	*P* value	Test statistics	*P* value
Physical limits	History of smoking (Yes)	—	—	3.424	0.001**^a^
Angina stability	TC (mmol/L)	-0.139	0.043*^b^	—	—
	HDL-C (mmol/L)	-0.148	0.031*^b^	—	—
	Hs-CRP (mg/L)	-0.171	0.012*^b^	—	—
Angina frequency	Gender (Male)	—	—	-3.654	<0.001**^a^
	Hypertension (Yes)	—	—	2.046	0.042*^a^
	T4 Anxiety (Yes) ^#^	—	—	-2.08	0.037*^a^
Treatment satisfaction	Implantable stent (Yes)	2.256	0.025*^a^	—	—
	T1 Anxiety (Yes) ^#^	-2.58	0.010* ^a^	—	—
	T2 Anxiety (Yes) ^#^	-2.17	0.030* ^a^	—	—
Disease perception	TG (mmol/L)	-0.152	0.027*^b^	—	—
	T3 Anxiety (Yes) ^#^	-2.21	0.027* ^a^	—	—

^a^Mann-whitney U test was used to analyze the relationship between SAQ dimensions and independent variables at 3 and 6 months after PCI.

^b^Pearson correlation was used to analyze the relationship between SAQ dimensions and independent variables at 3 and 6 months after PCI.

^**#**^ Less than 50 means no anxiety, and greater than or equal to 50 means anxiety [21, 22].

hs-CRP: high sensitivity C-reactive protein, TC: total cholesterol, TG: triglyceride, HDL-C: high-density lipoprotein cholesterol.

*P< 0.05.

**P< 0.01.

**Table 5:** Multivariate linear regression analysis of influencing factors on the treatment satisfaction dimensions of SAQ at 3 months and on the Anginal frequency dimensions of SAQ at 6 months after PCI.

Items	Treatment satisfaction dimensions of SAQ (3 months after PCI)				Anginal frequency dimensions of SAQ (6 months after PCI)			
	*B*	*t*	95%CI	*P* value	*B*	*t*	95%CI	*P* value
T1 Anxiety (YES)	-57.26	-2.97	(-28.35, -7.33)	0.002**	—	—	—	—
T2 Anxiety (YES)	-28.21	-2.53	(-15.05, -0.90)	0.024*	—	—	—	—
Implantable stent (YES)	18.21	2.56	(-15.31, -1.10)	0.031*	—	—	—	—
T4 Anxiety (YES)	—	—	—	—	33.76	2.28	(1.15,6.38)	0.005**
Gender (Male)	—	—	—	—	1.95	1.01	(1.28,3.62)	0.022*

*P< 0.05.

**P< 0.01.

**Table 6:** Relationship between readmission and quality of life at 3 and 6 months after PCI.

Items	3 months after PCI			6 months after PCI		
	Readmission group^a^	*Z*	*P* value^b^	Readmission group^a^	*Z*	*P* value^b^
Physical limits	62.22(48.89,78.33)	-0.334	0.738	77.78(57.78,80.00)	-1.087	0.277
Angina stability	100(75.00,100)	-0.878	0.380	75.00(75.00,100)	-0.235	0.814
Angina frequency	70.00(60.00,82.50)	-0.487	0.633	70.00(60.00,80.00)	-0.488	0.626
Treatment satisfaction	52.94(52.94,76.47)	-0.149	0.882	52.94(52.94,70.59)	-0.282	0.778
Disease perception	66.67(58.33,66.67)	-0.616	0.538	75.00(66.67,75.00)	-1.721	0.087
Total SAQ score	346.6(306.62,388.56)	-0.181	0.857	352.11(330.16,378.81)	-0.380	0.704

^a^ The values were given as the median and 25^th^ and 75^th^ percentile.

^b^ Mann-whitney U test was used to analyze the relationship between readmission and quality of life at 3 and 6 months after PCI.

### The relationship between peri-procedure anxiety and readmission rate

There were no statistically significant differences in gender, age, education level, employment, smoking history, history of hypertension, history of diabetes, family history of coronary heart disease, BMI, TC, TG, LDL-C, HS-CRP, LVEF (*P*>0.05) between readmitted patients and non-readmitted patients. Full details are given in Table 7. The results showed statistically significant differences between the anxiety and the readmission rate during the follow-up period in the patients’ pre-procedure 3 days (*χ*^*2*^ = 4.459, *P* = 0.001), pre-procedure 1 day (*χ*^*2*^ = 3.354, *P* = 0.001), and post-procedure 1 day (*χ*^*2*^ = -2.237, *P* = 0.026). See Table 8 for details. After adjusting for other confounding factors, the results of multiple logistics regression showed that T2 with anxiety (*B* = 1.445, *P* = 0.010, 95% CI: 1.409–12.773) and T4 without anxiety (*B* = -1.587, *P* = 0.042, 95% CI:0.044–0.941) were risk factors for readmission for cardiovascular reasons within half a year. The results are presented in Table 9. To further understand the relationship between anxiety fluctuations and readmission, we also analyzed the relationship between the average score of anxiety at 4 time points and readmission, and the results showed that the score of anxiety evaluation and readmission had statistical significance (*Z* = -2.08, *P* = 0.038). Full details are given in Table 10.

**Table 7:** Comparison of information on readmitted patients and non-readmitted patients.

Characteristic	Non-readmission group	Readmission group	Test statistics	*P* value
Gender				
Male	100(63.3)	36(66.7)	0.199	0.654^a^
Female	58(36.7)	18(33.3)		
Age (Years)				
<65	63(39.9)	18(33.3)	0.729	0.390^a^
≥65	95(60.1)	36(66.7)		
Education level				
Senior high school and above	12(7.6)	6(11.1)	2.982	0.749^a^
Junior high school and below	146(92.4)	48(88.9)		
Job				
Yes	38(24.1)	14(25.9)	0.076	0.783^a^
No	120(75.9)	40(74.1)		
Smoking history				
Yes	81(51.3)	28(51.9)	0.252	0.828^a^
No	77(48.7)	26(48.1)		
History of hypertension				
Yes	76(48.1)	27(50.0)	0.058	0.810^a^
No	82(51.9)	27(50.0)		
History of diabetes				
Yes	32(20.3)	9(16.7)	0.332	0.565^a^
No	126(79.7)	45(83.3)		
Family history of CHD				
Yes	4(2.5)	3(5.6)	1.153	0.309^a^
No	154(97.5)	51(94.4)		
Implantable stent				
Yes	90(57.0)	22(40.7)	4.250	0.042*^a^
No	68(43.0)	32(59.3)		
Type of drugs taken (type)				
≤3	93(58.9)	28(51.9)	1.186	0.526^d^
4–5	60(38.0)	25(46.3)		
≥6	5(3.1)	1(1.8)		
BMI (kg/m^2^)	23.67±3.11	24.46±3.98	-1.322	0.190^b^
TC (mmol/L)	4.43±0.90	4.30±0.87	0.932	0.352^b^
TG (mmol/L)	1.74±1.19	1.41±0.79	1.928	0.055^b^
HDL-C (mmol/L)	1.16±0.26	1.04±0.19	3.744	<0.001**^b^
LDL-C (mmol/L)	1.72±1.09	1.69±0.72	0.182	0.855^b^
LVEF(%)	62.01±9.01	60.81±8.90	0.842	0.401^b^
Hs-CRP (mg/L)	1.00(0.50,4.30)	1.05(0.58,4.10)	-0.439	0.660^c^

^a^ The Chi-square test was used to compare factors affecting readmission and non-readmission.

^b^ An independent sample T-test was used to compare factors influencing readmission and non-readmission.

^c^ The Mann-Whitney U test was used to compare factors influencing readmission and non-readmission.

^d^ The Fisher’s exact test was used to compare factors influencing readmission and non-readmission.

BMI: body mass index, LVEF: left ventricular ejection fraction, hs-CRP: high sensitivity C-reactive protein, TC: total cholesterol, TG: triglyceride, HDL-C: high-density lipoprotein cholesterol, LDL-C: low-density lipoprotein cholesterol.

*P< 0.05.

**P< 0.01.

**Table 8:** The relationship between peri-procedure anxiety and readmission.

Point of time(Anxiety)	Readmission (n = 54)^a^	*χ* ^*2*^	*P* value ^b^
T1(YES)	52 (25.2)	0.446	0.656
T2(YES)	35 (19.8)	6.459	0.001**
T3(YES)	30 (17.5)	6.354	0.001**
T4(YES)	51 (27.4)	3.937	0.026*

^**a**^ The values were given as the number of patients/implants with the percentage in parentheses.

^**b**^ Fisher accuracy test was used to analyze the relationship between peri-procedure anxiety and readmission rate.

*P< 0.05.

**P< 0.01.

**Table 9:** Logistic regression analysis of influencing factors on readmission rate.

Items	B	Exp(B)	95%CI	*P* value
Anxiety (T2)	1.45	4.242	(1.41,12.77)	0.010*
Anxiety (T4)	-1.59	0.205	(-0.04, -0.94)	0.042*
HDL-C	-2.07	4.181	(0.02,0.92)	0.041*
Implantable stent(YES)	0.99	4.270	(1.05,6.95)	0.039*

HDL-C: high-density lipoprotein cholesterol.

*P<0.05.

**Table 10:** The relationship between peri-procedure anxiety scores and readmission.

Items	Non-readmission group	Readmission group	*Z*	*P* value ^b^
Anxiety score	58.00(56.25,60.75)^a^	62.56(56.94,62.56)^a^	-2.08	0.038*

^a^ The values were given as the median and 25^th^ and 75^th^ percentile.

^b^ The Mann-Whitney U test was used to compare factors influencing readmission and non-readmission.

*P<0.05.

## Discussion

This study found that the anxiety level of patients with CHD fluctuated during the peri-procedure period of PCI and peaked 3 days before PCI, which was similar to the conclusion of Delewi’s study [25]. At the time of hospitalization appointment(T1), the patient was just diagnosed with CHD, they maybe mainly worried about the disease itself, such as what CHD is and what harm it has, etc. [26], the anxiety score was 57(55,61). Three days before the procedure(T2), the patients were waiting for the procedure at home, at this time, the date of PCI was getting closer and closer, they were not only worried about the disease itself, but also about PCI, such as the process of PCI, rehabilitation after PCI, and the cost of PCI [26]. So, the anxiety score increased to 64(61,68). The anxiety score of the first day before procedure(T3) was 54(51.58), which was lower than the anxiety level of T2, probably because the patient had already come to the hospital, had some communication with the medical staff, and had his or her doubts partially resolved, and was now probably mainly worried about PCI, such as worries about the outcome of the procedure, whether there will be accidents during the procedure, whether the procedure is painful, etc. [9]. Fourth, 1 day after PCI (T4), the procedure had been completed, and the main factor of anxiety of the patients turned to worry about the prognosis and the cost of follow-up treatment [27], and the overall anxiety level showed a downward trend.

A meta-analysis of 11 cross-sectional studies found that the incidence of anxiety after CHD was as high as 37% [28]. One study found that without timely psychological intervention, anxiety levels in even mild coronary artery disease patients did not decrease significantly after 8 weeks, and increased the incidence of somatization symptoms in patients, leading to an increase in the number of outpatient visits and readmissions [29]. The incidence of peri-procedure anxiety during PCI was higher than that in other studies [30], which may be due to the lower educational level of the patients included in this study and their less knowledge and understanding of the disease. Concerning the hospitalization process in domestic hospitals [31], patients with CHD in our hospital have to go to the ward to make an appointment for hospitalization, and then wait for the hospitalization notice at home, and are admitted to the hospital 1 day before the procedure, after 1–3 days of post-procedure observation, they can be discharged with medicine after no obvious procedural complications. Therefore, specialist nurses should monitor the patient’s condition and conduct psychological evaluation during the peri-procedure period. Online and offline activities can be organized to educate patients about disease knowledge, psychological management methods and matters for attention during the recovery period after PCI, so as to reduce the anxiety level of patients.

Our study indicates that the quality of life of patients gradually improved with longer time after PCI, which is generally consistent with the findings of Wijeysundera [32]. However, the score of TS dimension was low, which was only 52.94(52.94,64.71) at 6 months. In addition, the score of angina stability decreased from 100.00(75.00,100.00) at 3 months to 75.00(75.00,100.00) at 6 months, which was consistent with the results of Cohen [33]. Patients after PCI require more interventions than those with coronary artery bypass grafting and require long-term antianginal medications [34], which may be associated with lower treatment satisfaction. In this study, 75% of the patients did not have a job, 70% of the patients had a junior high school education or below, and their economic income was generally low. Long-term medication increased their economic burden. In addition, nearly 50% of the patients did not undergo stent implantation, and the angina symptoms did not improve significantly [35].

In this study, peri-procedure anxiety in patients with elective PCI was not significantly associated with total post-procedure quality of life scores, which is consistent with the findings of Chang et al. [36], whose study followed 80 patients with stable angina and found that anxiety at baseline was significantly associated with quality of life scores, and that the lower the anxiety score, the higher the quality of life score at 1 year post-procedure. Our study found that whether anxiety at time T1 and T2 was negatively correlated with TS dimensions 3 months after PCI, and whether anxiety at time T4 was negatively correlated with AF dimensions 6 months after PCI. On the one hand, the peri-procedure anxiety is closely related to short-term prognosis after PCI, and on the other hand, the quality of life associated with angina pectoris after PCI is also affected by other factors, such as post-procedure ischemia-reperfusion injury, in-stent restenosis, thrombosis, inadequate prevention and control of risk factors for CHD or other reasons. Although PCI was effective in improving blood perfusion and stenosis in patients, it did not alter the pathological course of atherosclerosis, and patients also developed angina and other pre-procedure symptoms. The results of this study remind medical staff to pay more attention to patients’ psychological conditions such as peri-procedure anxiety, so as to prevent CHD recurrence and consolidate the treatment effect.

In this study, the readmission rate of patients was about 25%, higher than the results of many domestic and foreign studies [37–39]. Readmissions due to hypertension accounted for 48.15% of total readmissions. This may be due to the fact that the implementation time of this study was in winter, and the cold weather resulted in blood pressure of patients increased, while the blood pressure of the elderly fluctuated greatly, leading to a higher readmission rate [40]. Half of the patients in this study had hypertension, and as shown in Table 8, more than one-third of the patients were admitted to hospital for exacerbation of hypertension, confirming our conclusion. Our study also found that the readmission rate of angina was 6.6%, which was lower than the 18.8% readmission rate of Smith’s team within 6 months after procedure [41], possibly because this study enrolled patients with ST-segment elevation acute myocardial infarction, whose condition was relatively serious.

In addition to these findings, our study found that patients with anxiety at T2 and without anxiety at T4 were independent risk factors of readmission for cardiovascular reasons. A domestic follow-up of 308 patients with CHD treated with elective PCI showed that the 1-year post-procedure readmission rate of patients in pre-procedure anxiety group was 3 times higher than that in non-anxiety group [42], indicating that pre-procedure anxiety was closely related to readmission for cardiovascular reasons. Coronary atherosclerosis is a chronic inflammatory disease, and anxiety is positively associated with inflammatory factor levels, which can accelerate the pathological process of coronary atherosclerosis and affect plaque stability, thereby increasing readmission rates [43]. In addition, several studies have reported that patients with coronary heart disease accompanied by anxiety are prone to negative coping styles, and patients’ inability to adhere to a healthy lifestyle after discharge (still smoking, drinking alcohol, failing to control weight, failing to adhere to exercise and exercise), failing to take medication correctly and regularly, and failing to come to the hospital for regular review are also associated with readmission [44–46]. It is suggested that medical staff should contact and communicate with patients by telephone, strengthen the online education for patients and their families, or distribute the instruction manual for diseases and procedure in advance, which is believed to be beneficial to reduce patients’ anxiety. However, the readmission rate of patients without post-procedure anxiety was about 4.878 times higher than that of patients with anxiety (Exp (B) = 0.205), as shown in Table 9, which was inconsistent with the results of a previous study [41]. Analysis of the causes of the above results may be due to appropriate post-procedure anxiety, leading to regular and even frequent outpatient follow-ups and active cooperation with medication and seeking medical help, thus reducing readmission and facilitating post-procedure recovery.

On this basis, this study suggests that specialist nurses should conduct the whole psychological assessment and management of patients, issue related disease information and education sheets in advance, or even ask the professional psychological consultants to intervene in advance, so as to reduce the anxiety level of the patients while waiting at home. At the same time, it is also necessary to emphasize the importance of changing the bad lifestyle and self-monitoring of the disease after PCI, so as to prevent the patients from becoming blindly optimistic psychology. In addition, strengthening psychological assessment and emotional management will be beneficial to reduce the rate of cardiovascular readmission. However, there are still limitations in our study. Firstly, the convenience sampling method was adopted to select the subjects, so the representation of samples may be poor. Secondly, due to the lack of manpower, material resources and scientific research funds, the anxiety score could not be continuously monitored after discharge. In the future, we will conduct multi-center, prospective studies to provide the best evidence.

## Conclusion

This study showed that the anxiety level of CHD patients fluctuated in the peri-procedure period, and the number of anxious patients was the largest at Time point T2. Anxiety at T1, T2 and T4 were independent risk factors for quality of life at 3 and 6 months after PCI, respectively. T2 and T4 anxiety were closely related to the cardiovascular readmission rate 6 months after PCI and were independent risk factors. Our study suggests that intervention should be strengthened at each time point in the peri-procedure period in the future to improve post-procedure rehabilitation effect.

## Supporting information

S1 File Protocol (Chinese). (DOCX)

S2 File Model consent form (Chinese). (DOC)

S3 File Files of data. (XLS)

S4 File Clinical trial registration. (DOC)

S5 File STROBE-checklist. (DOCX)

S6 File Supplementary tables. (DOCX)
