# Supplementary material for: Relationship between dynamic changes of peri-procedure anxiety and short-term prognosis in patients undergoing elective percutaneous coronary intervention for coronary heart disease: A single-center, prospective study
Source: PLoS One. 2022 Apr 1;17(4):e0266006. doi: 10.1371/journal.pone.0266006 (PMC8974971; doi:10.1371/journal.pone.0266006)
Supplement: S1 File — (DOCX) [file pone.0266006.s001.docx]

**1研究背景**

冠状动脉硬化性心脏病(Coronary Heart Disease, CHD)是冠状动脉血管狭窄或阻塞所引起的急性心肌缺血、缺氧性心脏病，简称“冠心病”^[1]^。近30年来，心血管(cardiovascular disease, CVD)已经成为全球做主要的死亡原因。据世界卫生组织(WHO)发布的《2018世界卫生统计报告》，2016年全球约1,790万人死于心血管疾病，占全球死亡总人数的31.4%，占慢性非传染性疾病死亡人数的44%，而CHD所致的死亡位列所有疾病导致死亡的首位^[2]^。根据美国的一项报告^[3]^，2013-2016年，美国有1.215亿的成年人患有心血管疾病，2016年CHD成为美国心血管疾病相关死亡的主要原因。而《中国心血管报告2018》概要推算，我国心血管现患病人数为2.9亿，其中CHD就有1100万，而CHD导致的城乡居民死亡率分别为113.46/10万，118.74/10万^[4]^。由此可见，CHD具有较高的发病率及死亡率，严重威胁着民众的健康，同时，CHD也一直是我国面临的一个重要的公共卫生问题。

目前CHD的治疗主要有药物治疗和手术治疗等。其中经皮冠状动脉介入治疗(Percutaneous Coronary Intervention, PCI)因损伤性小、痛苦轻、住院时间短等优点，现在已经成为CHD主要的治疗手段。根据手术时机不同，PCI可分为急诊PCI和择期PCI^[5]^。择期PCI是指无急诊PCI指征，结合患者病情特点合理安排其接受PCI的治疗策略，适用于稳定性心绞痛、中低危非ST段抬高型急性冠脉综合征患者^[6]^。尽管PCI可显著减轻冠心病症状、降低死亡率^[7]^,但是患者仍有可能发生主要心脏不良事件（Major Adverse Cardiovascular Events, MACE）的风险。Rao^[8]^的研究显示，行择期PCI治疗的老年稳定性心绞痛患者术后1个月的再入院率与死亡率分别为9.6%和0.22%。刘斌^[9]^的研究也指出，CHD患者支架植入术后发生支架内再狭窄的概率达5%-30%，给患者的治疗效果和预后造成极大的不利影响，增加了再入院率。

CHD患者的预后是由多种因素决定的。马士容^[10]^的研究显示，围手术期焦虑是影响PCI术后12个月MACE的独立影响因素，Frasure-Smith^[11]^等的研究指出，广泛性焦虑障碍与CHD患者2年MACE发生有关，Strik^[12]^等的研究发现，焦虑是心肌梗死患者发生心脏不良事件的独立危险因素。所以，只有控制好已知的危险因素，才能使CHD患者获益最大。焦虑是个体经历到一种不安、不舒适且不确定的感受，并且对此不可预知的威胁产生的自主神经系统上的反应，是人类普通的一种情绪反应^[13]^。Craske^[14]^等学者指出，当个体预感到潜在的危险或不幸时，很容易表现为持续性紧张。CHD患者常常因发病时的疼痛和不适以及对CHD认知不足等产生多种心理问题，其中焦虑最为常见[15]。韩冰^[16]^的调查结果显示，203例行PCI治疗的患者，术前焦虑发生率为22.2%，术后1周的焦虑发生率为9.9%，Xiao^[17]^等人对170例行PCI治疗的患者研究表明，术前1天焦虑的发生率为34.7%，术后1天的焦虑发生率为54.7%。由此可见，焦虑在PCI术前术后均可存在。

焦虑可导致体内交感神经兴奋性增加，儿茶酚胺水平升高，促凝物质大量释放，使心率加快，从而诱发或加重心绞痛、心肌梗死等心脏事件^[18]^。焦虑情绪对于心血管疾病的预后存在明确的预测作用。大量研究表明，焦虑障碍与冠心病互为因果、互相影响，冠心病患者容易合并焦虑情绪，焦虑情绪又严重影响冠心病患者的治疗及预后^[19-20]^，多项研究^[21-22]^还表明，合并焦虑行PCI治疗的冠心病患者MACE发生率显著高于心理正常患者。择期PCI治疗作为冠心病治疗的主要手段之一。临床研究发现，焦虑亦是冠心病患者行择期介入手术前后常见的情绪问题^[23]^，它可对手术效果和预后产生影响^[24]^，还可影响患者的生活质量^[25-26]^。据国内外文献报道，术前焦虑不仅给患者带来心理痛苦，而且会干扰手术的顺利进行，从而消极地影响治疗效果，甚至造成术后并发症的发生^[27-28]^。多数患者围术期常易出现较大的情绪波动，尤其是负面心理应激，导致免疫功能紊乱，影响患者术后康复^[29]^。

目前学者对冠心病焦虑的研究时间点多集中于术前或术后某一时间点。患者预约后至术前等候期，也是冠心病患者择期手术治疗全过程的重要组成部分之一，很多患者在此期间会出现度日如年的心理，他们的焦虑水平如何，对其预后是否有影响，国内外未见报道。如何缓解因手术而产生的焦虑，提高患者的心理应对能力正越来越受到关注。故针对上述情况，有必要对CHD患者介入治疗围手术期焦虑状态进行动态监测，了解CHD患者围手术期焦虑的现状与术后心绞痛相关生活质量及再住院的关系，为CHD患者围手术期焦虑情绪障碍的干预提供理论及实践依据。

**2研究目的及意义**

了解冠心病（Coronary artery heart disease，CHD）择期介入手术患者行PCI围手术期的焦虑、术后生活质量和因心血管原因再入院率现状，分析围手术期焦虑对术后生活质量和因心血管原因再入院率的影响，为CHD患者围手术期焦虑情绪障碍的干预提供理论及实践依据。

**3概念界定**

**3.1经皮冠状动脉介入术：**是指在冠状动脉造影的基础上运用导管技术，通过球囊加压扩张、机械切割、激光气化、放置金属支架等方法，使狭窄的冠脉内径扩大，从而改善心肌血液供给，缓解患者临床症状的治疗手段^[30]^。

**3.2择期PCI术**：患者入院后常规给予营养心肌、扩充冠状动脉等支持治疗，同时给予低分子肝素 6000U皮下注射，2次/d，氯吡格雷75mg、阿司匹林100mg，1次/d，术前进行心脏超声检查，并对心功能进行评估，一般在入院后或发病后7～14d 进行PCI治疗^[31]^。

**3.3焦虑：**是指人们内心的一种不安状或无来由的恐惧感，尤其当个体预感到潜在的危险或不幸时，容易表现为持续性精神紧张^[14]^。

**3.4围手术期：**是指围绕手术的一个全过程，从病人决定手术治疗开始，到手术治疗结束，包括手术前、手术中、手术后的一段时间^[32]^。本研究中围手术期是指PCI术前一周至术后出院的一段时间。

**3.5生活质量**：世界卫生组织(World Health Organization，WHO)将生活质量(Quality oflife)定义为个体在其所生活的文化和价值体系背景下，对其目标、期望、标准和关切事项有关的生活状况的感知^[33]^。本研究主要涉及冠心病患者的生活质量，故用西雅图心绞痛量表评估而得。

**3.6西雅图心绞痛评分：**由西雅图心绞痛量表(Seattle angina questionnaire，SAQ)评估而得，是针对冠心病心绞痛患者生活质量的特异性量表，此表有19个条目；5个维度即躯体活动受限程度，心绞痛稳定状态，心绞痛发作情况，治疗满意程度，疾病认知程度。得分越高，患者生活质量及机体功能状态越好^[34]^。

**3.7再入院：**是指住院患者在出院一段时间后又因相同的或相关的疾病再次住院；相同的疾病指的是上次出院主要诊断与再次住院的主要诊断相同；相关疾病是指再次入院的疾病与上次入院的疾病有关联，包括计划性再住院和非计划性再住院；计划性再住院是指在上次住院过程中，医患双方共同制定的治疗方案计划在患者出院一段时间后重新住院接受进一步治疗的过程；非计划再住院，是指患者上次住院诊疗结束后，因同一疾病或相关疾病发生未计划的、不能预测的再次住院^[35]^。本研究主要涉及相关疾病的非计划性再住院。

**3.8短期预后：**指冠心病患者PCI治疗后3个月或6个月后出现的主要心脏不良事件（MACE）的情况^[36]^。本研究主要收集PCI术后3至6个月的生活质量及6个月内的再入院情况。

**参考文献**

1. 国家卫生计生委合理用药专家委员会,中国药师协会.冠心病合理用药指南[J].中国医学前沿杂志(电子版),2016,8(6):19-108.
2. Organization WH.World health statistics 2018.Monitoring health for the SDGs Sustainable Development Goals.[J].Geneva,Switzerland,WHO,2018.
3. Benjamin EJ,Muntner P,Alonso A,et al.Heart Disease and Stroke Statistics-2019 Update:A Report From the American Heart Association. Circulation,2019,139(10):e56-e528.
4. 胡盛寿,杨跃进,郑哲,等.《中国心血管病报告2018》概要[J].中国循环杂志,2019,34(3):209-220.
5. 于学忠,张新超,朱华栋,等.急性冠脉综合征急诊快速诊疗指南[J].中华危重症医学杂志(电子版),2016,36(2):207-214.
6. 韩雅玲.PCI围术期抗血栓治疗的再思考—《中国PCI指南(2016)》解读[J].中华保健医学杂志,2016,18(5):349-351.
7. Serruys P W,Morice M C,Kappetein A P,et al.Percutaneous Coronary Intervention versus Coronary-Artery Bypass Grafting for Severe Coronary Artery Disease[J]. New England Journal of Medicine,2009,360(10):961-972.
8. Rao SV,Kaltenbach L A,Weintraub W S,et al.Prevalence and Outcomes of Same-Day Discharge After Elective Percutaneous Coronary Intervention Among Older Patients[J]. Jama,2011,306(13):1461-1467.
9. 刘斌,陶贵周.冠状动脉内支架置入术后支架内再狭窄的相关因素分析[J].中国循环杂志,2017,32(z1):92.
10. 马士容.PCI围术期舒适护理对冠心病患者心理状态及预后的影响[J].中华全科医学,2015,13(11):1866-1868.
11. Frasure-Smith N,Lespérance,Franrois.Depression and Anxiety as Predictors of 2-Year Cardiac Events in Patients With Stable Coronary Artery Disease[J]. Archives of General Psychiatry,2008,65(1):62-71.
12. Strik JJ,Denollet J,Lousberg R,et al.Comparing symptoms of depression and anxiety as predictors of cardiac events and increased health care consumption after myocardial infarction[J]. journal of the american college of cardiology,2003,42(10):1801-1807.
13. 李乐之,Wanpen Eamjoy.腹部手术患者术前焦虑与社会支持的相关性研究[J].护士进修杂志,2000,15(3):171-174.
14. Craske MG,Stein MB,Eley TC,et al.Anxiety disorders[J].nature reviews disease primers,2017,3:17024.
15. Lynn VD,Debra KM,Barbara Riegel,et al.Persistent comorbid symptoms of depression and anxiety predict mortality in heart disease[J].International Journal of Cardiology,2010,145(2):180-192.
16. 韩冰.冠心病患者PCI围术期焦虑、抑郁状态评估及影响因素分析[D]．大连：大连医科大学,2018.
17. Xiao Y,Zhou J,Zheng J,et al.Impact of depression and/or anxiety on patients with percutaneous coronary interventions after acute coronary syndrome: a protocol for a real-world prospective cohort study[J]. Bmj Open, 2019, 9(9):e027964.
18. Goldstein BI,Carnethon MR,Matthews KA,et al.Major Depressive Disorder and Bipolar Disorder Predispose Youth to Accelerated Atherosclerosis and Early Cardiovascular Disease:A Scientific Statement From the American Heart Association[J]. Circulation,2016,132(10):965-986.
19. Celano CM,Millstein RA,Bedoya CA,et al.Association between anxiety and mortality in patients with coronary artery disease:A meta-analysis[J].American Heart Journal,2015,170(6):1105-1115.
20. 余灿清,陈怡平,吕筠,等.中国30-79岁成人抑郁障碍与冠心病和脑卒中的关联[J].北京大学学报:医学版,2016,48(3):465-471.
21. 王海珍,江庆,伍万仕,等.负性情绪对冠心病 PCI 手术患者预后的影响[J].心血管康复医学杂志,2015,(2):130-133.
22. 陈烨,朱晓燕,刘洪珍,等.团体咨询联合认知行为疗法对老年冠心病患者PCI术后情绪与心脏不良事件发生率的影响[J].中华现代护理杂志,2018,24(1):64-70.
23. 李芬.冠心病患者择期介入治疗前后焦虑抑郁的心理干预[J].心血管康复医学杂志,2012,21(4):358-360.
24. 李永斌,姚朱华,许振坤,等.冠心病患者介入术后焦虑抑郁情绪的变化及影响因素[J].中华医学杂志,2012,92(21):1498-1501.
25. 谭燕,余韬,段春枝,等.双心护理对择期冠心病PCI后患者病情管理能力及生活质量的影响[J].现代医药卫生,2018,34(11):1625-1628.
26. 李金鹤,张艳春,马妍.冠心病介入术患者围手术期心理干预的研究[J].中国循环杂志,2014,(z1):210-210.
27. 郑舒.骨科手术患者焦虑评估及心理护理[J].实用医学杂志,2011,27(2):315-316.
28. Auyeung K,Hawley LL,Grimm K,et al.Fear of Negative Evaluation and Rapid Response to Treatment During Cognitive Behaviour Therapy for Social Anxiety Disorder[J].Cognitive Therapy and Research,2020(2):147-154.
29. 宋爱胜,万效梅.循证护理对冠心病择期手术患者术前焦虑状态的影响[J].中华现代护理杂志,2014,17(1):79-81.
30. Colleran R,Kastrati A.Percutaneous coronary intervention:balloons,stents and scaffolds[J].Clinical Research in Cardiology, 2018,107(S2):55–63.
31. 古平,潘波,牟海刚.急诊PCI与择期PCI治疗急性心肌梗死的临床对比分析[J].现代仪器与医疗,2014(2):48-50,82.
32. Thompson BM,Stearns JD,Apsey HA,et al.Perioperative Management of Patients with Diabetes and Hyperglycemia Undergoing Elective Surgery[J].Curr Diab Rep.2016,16(1):2.
33. The World Health Organization Quality of Life assessment (WHOQOL): position paper from the World Health Organization[J]. Soc Sci Med. 1995;41(10):1403–1409.
34. 刘淑红.西雅图心绞痛量表(SAQ)文译本的信度、效度、反应度[D].天津医科大学,2003.
35. 杨辉.再入院:概念、测量和政策意义[J].中国卫生质量管理,2009,16(5):113-115.
36. 吴苑苑.不同时间窗择期PCI对急性心肌梗死患者短期预后的影响[D].河南科技大学,2019.

**4研究对象及方法**

**4.1研究对象**

采取便利取样法，选取2019年12月至2020年12月在常熟市第一人民医院心内科行择期介入手术的220例冠心病患者为研究对象。于2019年12月30日招募第一例受试者。**纳入标准**：①年龄≥18岁；②诊断符合《内科学(第九版)》中关于冠心病的诊断标准^[37]^的冠心病患者，包括稳定型心绞痛、不稳定型心绞痛和心肌梗死的患者；③根据病情需要进行择期冠脉介入手术者；④愿意签署知情同意书。**排除标准**：①有冠心病介入手术史；②近期存在急性感染者；③重要脏器衰竭难以行介入术者；④有精神病或认知障碍难以配合者；⑤无法或者拒绝完成随访者。

**4.2样本量**

根据探索有关变量的影响因素研究的样本量要求，样本量至少是变量数目的15～20倍的原则，通过文献回顾，本研究约有10个影响变量，考虑到20%的样本流失率，预计样本量为180-240例，本研究最终纳入220例。

**4.3研究工具**

4.3.1一般资料问卷

该问卷是在查阅文献的基础上由研究团队自行设计，包括年龄、性别、教育程度、在职情况、吸烟史等。

4.3.2临床资料

包括支架植入与否、左室射血分数、超敏C反应蛋白、总胆固醇、甘油三酯、高密度脂蛋白胆固醇、低密度脂蛋白胆固醇、冠心病家族史、高血压病史、糖尿病史等。左心室射血分数的检测：入院24h内由专业的心超室医师完善超声心动图（PHILIPS5500型多普勒超声仪，探头频率为2.5Hz）检查，测量左心室射血分数（Left ventricular ejection fraction，LVEF）。

4.3.3焦虑自评量表(Self-Rating Anxiety Scale，SAS)

由华裔教授Zung编制(1971)^[38]^。按照中国常模结果^[39]^，SAS标准分的分界值为50分，其中50-59分为轻度焦虑，60-69分为中度焦虑，69分以上为重度焦虑。总分评定的信度系数为0.93，效度系数为0.86，本研究中Cronbach's α系数是0.82。

4.3.4西雅图心绞痛量表评分（Seattle Angina Questionnaire，SAQ）

由美国学者Spertus于1994年研制，是针对冠心病患者特定的功能状态及生活质量的量表。量表共19个条目，分为5个维度：躯体活动受限程度（physical limitations，PL）9个条目、心绞痛稳定状态（anginal stability，AS）1个条目、心绞痛发作情况（anginal frequency，AF）2个条目、治疗满意程度（treatment satisfaction，TS）4个条目、疾病认识程度（disease perception，DP）3个条目。每个条目计分1~6分。维度得分=该维度条目的实际得分相加获得，然后再将实际得分按下面公式转化成标准得分，公式为：标准分（%）=(实际得分-该维度最低分) ×100/(该维度最高分-该维度最低分)。评分越高，表示患者生活质量及机体功能状态越好。此量表由国内学者刘淑红^[34]^汉化，具有较好的信效度，其中5个维度的内部一致性Cronbach’s α系数分别为0.914、1.000、0.837、0.804、0.607，5个维度的重测信度相关系数分别为0.982、0．953、0.979、0.966、0.943、0.984。该工具的内部一致性Cronbach’s α系数0.8，重测系数0.913，本研究中Cronbach's α系数是0.85。

4.3.5 心血管原因再入院率

本研究中，再入院率界定为因心血管问题引起的再入院，包括：高血压、心绞痛、心律失常、心功能不全等，排除感冒、肿瘤等其他因素而再入院的病例。由研究者电话随访收集随访6个月中的再入院情况。

**4.4资料收集**

4.4.1收集人员及时间

所有调查员均为心血管内科专业技术人员（包括研究者本人、2名中级以上护理人员、1名护理研究生、1名心内科副高职称医生），并由研究者本人对调查员进行统一培训，使各成员能够掌握调查的目的及过程，保证收集方式统一，以减少信息偏倚。

由于我院择期PCI术从预约至手术平均时间为1周，故收集围手术期焦虑资料时，将预约入院登记时作为T1，在家等候期（约术前3d）为T2，术前1d入院完善术前准备时为T3，术后1d为T4。主要研究者应用焦虑自评量表在上述时间点分别收集患者的焦虑资料；一般资料和临床资料于入院当天由研究团队完成收集；研究团队应用西雅图心绞痛评分问卷于术后3个月、6个月收集患者生活质量资料。术后6个月由主要研究者电话随访收集患者再入院资料。

4.4.2资料录入与整理

所有数据由2名护理研究生双人双机采用Epidata 3.1软件录入，分析数据阶段咨询专业统计学专家，从而保证分析结果的准确性。

**4.5资料收集与管理**

研究人员向患者说明研究目的后，于入院登记时（T1）、术后1d（T4）在病区示教室发放焦虑自评量表，指导患者或家属当场填写回收；术前1d（T3）在病房示教室发放一般资料问卷，由患者或家属填写一般资料，研究团队成员通过“德朗诺医疗信息平台”查询患者的临床资料，以完善问卷，并指导填写焦虑问卷；术前3d（T2）通过电话随访询问患者焦虑情况并填写焦虑自评量表；术后3个月、6个月由医生行电话随访，询问患者躯体活动受限程度、心绞痛稳定状态、心绞痛发作等情况，填写西雅图心绞痛量表。术后6个月由主要研究者电话随访收集患者术后半年内有无再入院及再入院的原因。

**4.6统计学方法**

1. 符合正态分布的计量资料采用（‾x ±s）表示，两组间比较用*t*检验；
2. 计数资料用n（%）表示，组间比较用卡方检验或Fisher’s精确检验；
3. 多因素线性回归分析患者围手术期焦虑对生活质量的影响，多因素Logistic回归分析患者围手术期焦虑对再入院率的影响；
4. 双侧*P*＜0.05表示差异有统计学意义。

**4.7技术路线图（见下页）**


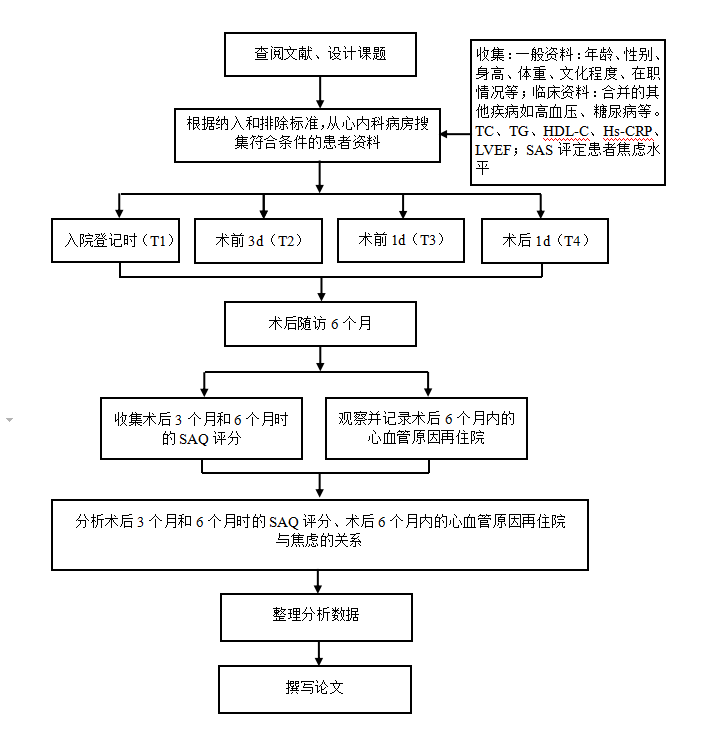


**5 研究意义**

本研究通过了解冠心病（Coronary artery heart disease，CHD）择期介入手术患者行PCI围手术期的焦虑、术后生活质量和因心血管原因再入院率现状，分析围手术期焦虑对术后生活质量和因心血管原因再入院率的影响，可以为CHD患者围手术期焦虑情绪障碍的干预提供理论及实践依据。

**6预期进展及研究成果**

（1）通过本项研究，为CHD患者围手术期焦虑情绪障碍的干预提供理论及实践依据。

（2）发表核心或统计源期刊论文0～1篇。

（3）参加国内学术会议交流1次。

**参考文献**

1. 葛均波,徐永健,王辰.内科学(第九版)[M].人民卫生出版社, 2018.
2. Zung WWK.A Fating instrument for Anxicty Disorders,Psychosomatics, 1971, 12:371-379.
3. 汪向东,王希林,马弘.心理卫生评定量表手册(增订版)[M].北京:中国心理卫生杂志社,1999:235-237.

**7研究基础**

7.1 项目主要负责人学习与工作经历：

（1）2017.09-2020.06 苏州大学护理学院 硕士研究生

（2）2009.09-至今 常熟市第一人民医院心内科 主管护师

7.2 项目组主要成员科研及获奖情况

(1)苏州市科技局民生科技项目（SYS2018018），自主呼吸锻炼对稳定性冠心病患者心率变异性和心率收缩压乘积的效果研究，2018-06至2021-6，在研；

(2)苏州市护理学会，护理科研课题（A类），心力衰竭患者营养素摄入、营养风险现状及其与心脏无事件生存时间的相关性研究，2018.12至2019.12，已结题；

(3)苏州市科技局，民生科技项目（指导性），SYSD2016087，基于高危因素的健康管理对无症状颈动脉斑块患者的效果研究，2016.11至2017.11，已结题；

(4)Effects of transitional health management on adherence and prognosis in elderly patients with acute myocardial infarction in percutaneous coronary intervention:A cluster randomized controlled trial,PLOS ONE,2019,14(5):e0217535.

(5)自主呼吸锻炼对稳定性冠心病患者心率变异性和心肌氧耗的影响,中国老年学杂志,2019,39(5):1034-1037.

(6)冠脉支架植入术后患者过渡期自我护理体验的质性研究,中华护理杂志,2014,49(2):147-150.

(7)冠脉内支架植入患者过渡期生存质量及影响因素的研究,中国实用护理杂志,2013,29(27):30-34.

(8)苏州市自然科学优秀学术论文三等奖,苏州市人民政府,2016.

(9)专利：发明一项、实用新型专利5项。

**8经费预算**

| **科 目** | **申请经费** | **备注（计算依据与说明）** |
| --- | --- | --- |
| 设备购置费 | **100** | **签字笔** |
| 能源材料费 |  |  |
| 试验外协费 |  |  |
| 资料、印刷费 | **1000** | **调查表、文献打印** |
| 租赁费 |  |  |
| 差旅费 |  |  |
| 鉴定、验收费 |  |  |
| 管理费 |  |  |
| 其他费用 | **3000** | **论文发表费和其他不可预见的意外支出** |
| 合计 | 4100 | - |
