# Supplementary material for: Relationship between dynamic changes of peri-procedure anxiety and short-term prognosis in patients undergoing elective percutaneous coronary intervention for coronary heart disease: A single-center, prospective study
Source: PLoS One. 2022 Apr 1;17(4):e0266006. doi: 10.1371/journal.pone.0266006 (PMC8974971; doi:10.1371/journal.pone.0266006)
Supplement: S2 File — (DOC) [file pone.0266006.s002.doc]

**知情同意书**

病友朋友：

您好！我们现正进行“冠心病择期介入手术患者围术期焦虑水平与短期预后的关系”的调查研究，特邀请您参加。为了帮助您更好的做出选择，我们将本次研究的主要的目的及意义告知如下：

本研究的目的是分析冠心病患者择期介入治疗围手术期焦虑评分以及其对心血管原因再住院的影响,为CHD患者围手术期焦虑情绪障碍的干预提供理论及实践依据，以满足患者围术期的心理需求，降低患者再入院率。

如果您愿意参加此次的研究，我们将告知您以下内容：

1.在本次研究的过程中如涉及到您的个人资料，我们将根据国家有关规定严格保密，不向任何第三方透露。

2.您是否参加此次研究，不会给您的身体带来任何影响，我们仍将按原计划对您进行护理。

3.即使您已签订了知情同意书，您仍有权利在任何时间退出，而不会对您造成任何的影响

我真诚地希望您能同意参加本研究的资料收集，您所提供的资料对我们的研究非常重要。如果您有困难，您有权拒绝，或在任何时候退出。再次感谢您的参与。

祝身体健康，家庭幸福！

研究者：蔡雅静

年 月 日

**知情同意书**

调查员已向我介绍了该课题的目的和过程，我也知道了研究对我的身体、治疗和护理没有伤害。

我同意参加本课题的资料收集过程。签名：

日期 年 月 日
