# Supplementary figures and images for: Relationship between dynamic changes of peri-procedure anxiety and short-term prognosis in patients undergoing elective percutaneous coronary intervention for coronary heart disease: A single-center, prospective study
Source: PLoS One. 2022 Apr 1;17(4):e0266006. doi: 10.1371/journal.pone.0266006 (PMC8974971; doi:10.1371/journal.pone.0266006)

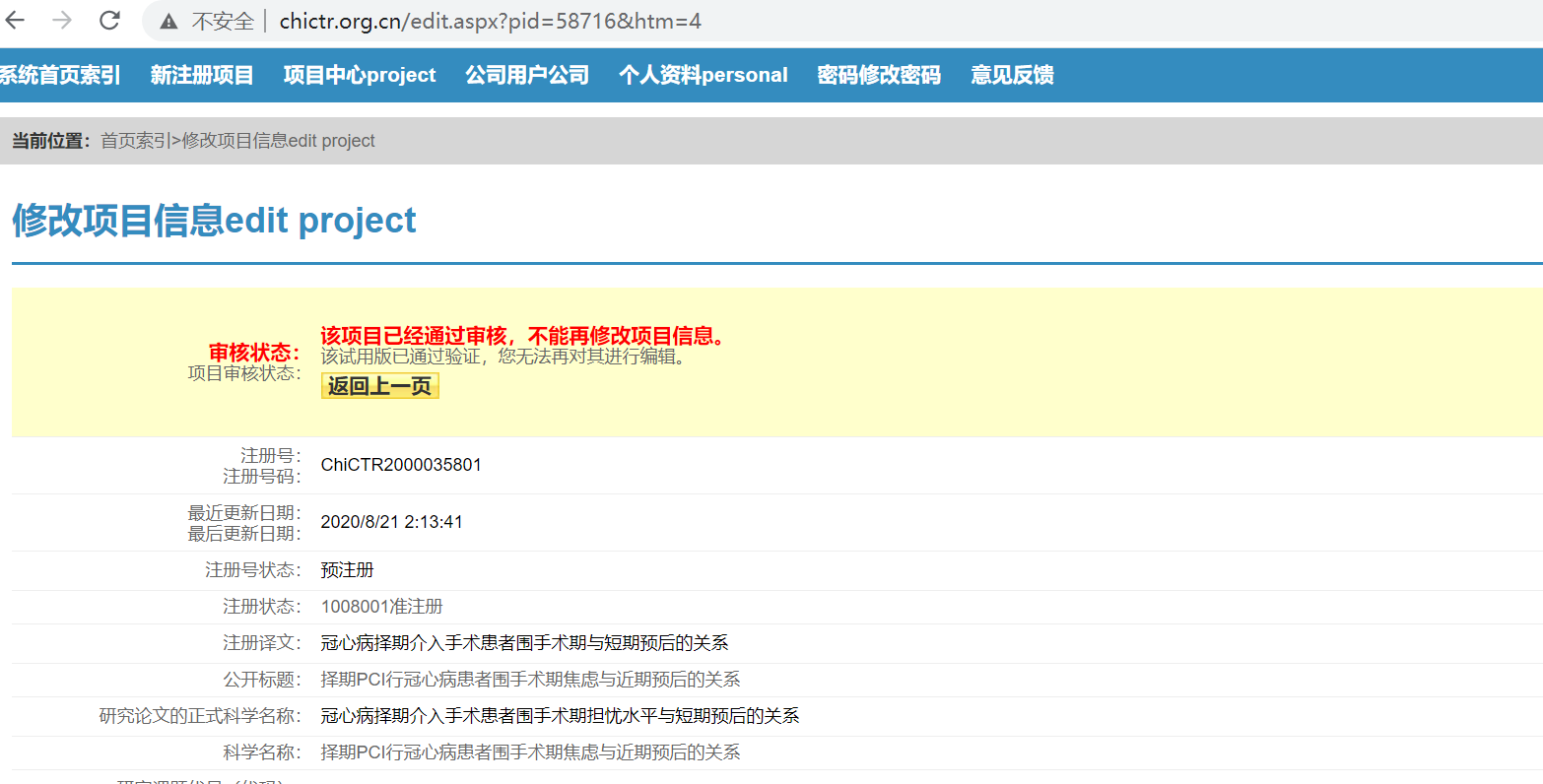

Supplement: S4 File — (DOC) [file pone.0266006.s004.doc]
