# Supplementary material for: Relationship between dynamic changes of peri-procedure anxiety and short-term prognosis in patients undergoing elective percutaneous coronary intervention for coronary heart disease: A single-center, prospective study
Source: PLoS One. 2022 Apr 1;17(4):e0266006. doi: 10.1371/journal.pone.0266006 (PMC8974971; doi:10.1371/journal.pone.0266006)
Supplement: S6 File — (DOCX) [file pone.0266006.s006.docx]

**The relationship between readmission adverse cardiovascular events and SAQ 6 months after PCI**

| **SAQ Dimension** | **Physical limits** | **Angina stability** | **Angina frequency** | **Treatment satisfaction** | **Disease perception** | **Total SAQ score** |
| --- | --- | --- | --- | --- | --- | --- |
| **hypertension**  **(n=26)^a^** | 77.78(57.78,80.00) | 75.00(75.00,100.00) | 70.00(60.00,80.00) | 52.94(52.94,66.18) | 75.00(64.59,75.00) | 352.11(332.75,387.26) |
| ***Z*** | -1.707 | -0.073 | -0.746 | -0.752 | -0.821 | -0.523 |
| ***P* value^b^** | 0.088 | 0.942 | 0.456 | 0.452 | 0.412 | 0.601 |
| **Angina**  **(n=14)^a^** | 66.67(57.78,77.78) | 100.00(75.00,100.00) | 70.00(60.00,82.50) | 58.83(52.94,69.12) | 75.00(66.67,75.00) | 361.56(340.47,385.72) |
| ***Z*** | -0.609 | -1.564 | -0.599 | -0.280 | -0.888 | -1.285 |
| ***P* value^b^** | 0.543 | 0.118 | 0.549 | 0.779 | 0.374 | 0.199 |
| **Arrhythmia**  **(n=8)^a^** | 56.67(48.89,63.33) | 100.00(62.50,100.00) | 70.00(62.50,87.50) | 50.00(35.29,70.59) | 62.50(43.75,72.92) | 306.30(290.78,367.43) |
| ***Z*** | -1.732 | -0.220 | -0.209 | -1.622 | -1.168 | -1.757 |
| ***P* value^b^** | 0.083 | 0.826 | 0.834 | 0.105 | 0.243 | 0.079 |
| **Cardiac insufficiency**  **(n=6)^a^** | 66.78(51.67,78.89) | 87.50(75.00,100.00) | 70.00(60.00,75.00) | 50.00(29.41,63.02) | 70.84(66.67,75.00) | 329.79(313.92,366.60) |
| ***Z*** | -0.123 | -0.289 | -0.775 | -1.352 | -1.398 | -0.683 |
| ***P* value^b^** | 0.902 | 0.773 | 0.450 | 0.176 | 0.162 | 0.494 |

^a^ The values were given as the median and 25^th^ and 75^th^ percentile.

^b^ Mann-whitney U test was used to analyze the relationship between various reasons for readmission and SAQ 6 months after PCI
